# Clinical Evaluation of Safety and Efficacy of a Central Current Good Manufacturing Practices Laboratory Produced Autologous Adipose-Derived Stromal Vascular Fraction Cell Therapy Product for the Treatment of Knee Osteoarthritis

**DOI:** 10.1089/scd.2024.0008

**Published:** 2024-04-03

**Authors:** Christopher J. Rogers, Robert Harman, Mitchell B. Sheinkop, Peter Hanson, Mary A. Ambach, Tal David, Rahul Desai, Steven Sampson, Danielle Aufierro, Jay Bowen, Gerard Malanga

**Affiliations:** ^1^ San Diego Orthobiologics Medical Group, Carlsbad, California, USA.; ^2^ Personalized Stem Cells, Poway, California, USA.; ^3^ Cellular Orthopedics, Chicago, Illinois, USA.; ^4^ Grossmont Orthopedic Medical Group, La Mesa, California, USA.; ^5^ Synergy Orthopedics Specialist Group, San Diego, California, USA.; ^6^ Restore PDX, Beaverton, Oregon, USA.; ^7^ Ortho Healing Center, Los Angeles, California, USA.; ^8^ New Jersey Regenerative Institute, Cedar Knolls, New Jersey, USA.

**Keywords:** adult stem cells, mesenchymal stem cells, knee, osteoarthritis, stromal vascular fraction, intra-articular injection

## Abstract

Knee osteoarthritis (KOA) is a prevalent condition characterized by the progressive deterioration of the entire joint and has emerged as a prominent contributor to disability on a global scale. The nature of the disease and its impact on joint function significantly limit mobility and daily activities, highlighting its substantial influence on patients' overall well-being. Stromal vascular fraction (SVF) is a heterogenous, autologous cell product, containing mesenchymal stem cells, derived from the patient's subcutaneous adipose tissue with demonstrated safety and efficacy in the treatment of KOA patients. We conducted a single-arm, open-label, multisite, FDA approved clinical study in Kellgren–Lawrence severity grade 2–4 KOA patients. The cellular product was manufactured from patient-specific lipoaspirate in a centrally located FDA-compliant manufacturing facility. Twenty-nine subjects were treated with a quality tested single intra-articular injection of GMP manufactured SVF. Adverse events, laboratory values, vital signs, and physical examination findings were monitored during the study period. Robust tolerability, without any substantial safety issues, was demonstrated. Knee pain and function, assessed through the Knee Injury and Osteoarthritis Outcome Score (KOOS), demonstrated notable improvements. These positive benefits persisted for up to 12 months, and the majority of participants expressed satisfaction. SVF from each patient was stored in a liquid nitrogen freezer for future clinical treatments. Unique to this study of autologous cells is the shipment of lipoaspirate from the clinic to a central FDA-compliant manufacturing facility for cleanroom-controlled manufacturing. The cell product characterization data demonstrate that this method produces an equivalent product in terms of cell count and viability with the added benefit of further quality assurance testing, including sterility, endotoxin, and flow cytometry, before patient administration. Clinical Trial Registration Number: NCT 04043819.

## Introduction

Arthritis is a leading cause of disability in the United States with annual costs for medical care and lost earnings exceeding $300 billion (https://www.cdc.gov/chronicdisease/resources/publications/factsheets/arthritis.htm). The most common form of arthritis is osteoarthritis (OA) with knee osteoarthritis (KOA) accounting for more than 80% of the burden of this disease [1,2]. Due to an aging population and rising rates of obesity, the number of Americans with arthritis is projected to increase to 78.4 million within the next 20 years [3].

Osteoarthritis is a progressive disease involving the entire joint [4]. An imbalance of anabolic and catabolic pathways leads to cartilage degeneration, synovial inflammation, and subchondral bone remodeling abnormalities. The ability of chondrocytes to self-renew diminishes with age [5]. Increased expression of inflammatory cytokines IL-1 β, TNF-α, and matrix metallopeptidase-13 [6] promotes the production of other pro-inflammatory factors, including IL-8, IL-6, leukotriene inhibiting factor, proteases, and prostaglandin E2. This array of inflammatory compounds leads to extracellular matrix degradation and the development of osteoarthritis [7].

There are currently no cures for OA. Symptoms are generally managed with a combination of weight loss, physical therapy, bracing, nonsteroidal anti-inflammatory medications (NSAIDS), analgesic medications, and injections of corticosteroids, hyaluronic acid, or platelet rich plasma [8]. These treatments often do not provide lasting improvement, changes in structural abnormalities, or restoration of function. In severe cases, total knee arthroplasty (TKA) is often recommended, but many patients are too young, have medical contraindications, or simply prefer to avoid surgery. It has been estimated that 3.6 million Americans suffer with pain and limited mobility without an effective treatment [9].

Adipose tissue has become important to the study of regenerative medicine in orthopedics [10]. Interest in adipose tissue as a source of cells useful for tissue repair has grown due to the abundance and availability of adipose through the safe and simple method of lipoaspiration [11,12]. Perivascular mesenchymal stem cells (MSCs) also known as “medicinal signaling cells” [13–15] have been identified in bone marrow, umbilical cord, skeletal muscle, and synovial tissue, but subcutaneous adipose tissue is the richest source of these regenerative cells [16]. Only a small portion of the nucleated cells derived from bone marrow aspirate (0.001–0.01%) consists of MSCs [17,18]. Whereas, in adipose tissue, up to 15%–30% may consist of MSCs (an adipose MSC commonly termed adipose stem or stromal cells, ASCs) [19] and are less likely to be influenced by a patient's age [20].

When ASCs are exposed to the synovial fluid from patients with KOA, their immunomodulatory properties are enhanced with reduced T cell proliferation and the generation of T regulatory cells [21,22]. Bioinformatics analysis of soluble factors and extracellular vesicles secreted by ASCs following exposure to an OA patient's synovial fluid provides the molecular basis for immunomodulation and cartilage protection in the osteoarthritic joint [23]. Long-term systemic immunomodulatory effects have also been demonstrated following intra-articular ASC administration [24]. These paracrine effects establish a regenerative microenvironment which promotes endogenous stem cell recruitment, activation, and differentiation [25].

The stromal vascular fraction (SVF) derived from adipose tissue is an abundant source of regenerative cells, including ASCs, pericytes, endothelial progenitor cells, macrophages, lymphocytes, fibroblasts, and smooth muscle cells [26–30], and obtained through enzyme-digested lipoaspirate without the need for culture expansion [16]. Standardized definitions of adipose stem cells and SVF have been proposed by the International Federation of Adipose Therapeutics (IFATS) and International Society of Cellular Therapy (ISCT) [26].

VetStem (VSB), the parent company and contract manufacturer for the Sponsor, has provided manufacturing for adipose-derived cell therapy for veterinarians since 2003 using a centralized FDA-compliant manufacturing model where adipose samples are collected in clinics and shipped refrigerated to a central laboratory for processing, storage, and return to clinics. Generally, PSC-01 is an enzymatically separated SVF similar to the originally published methods in the human [31] and utilized by the VSB facility in its canine and equine osteoarthritis research and development and clinical therapy programs as described in published studies [32,33].

The safety and efficacy of SVF cells have been previously reported in many human clinical trials [34–50]. Based on scientific and empirical evidence, we conducted an open-label, multisite, prospective clinical study designed to evaluate the safety of an intra-articular injection of autologous, adipose-derived SVF cells in patients with moderately severe KOA.

Unique to this clinical trial for autologous cells is this centralized FDA-compliant manufacturing method (current Good Manufacturing Practices, cGMP). Generally, in autologous SVF treatment publications, the method for adipose processing to SVF is using a point-of-care model and device where the cells are administered in the clinic without benefit of laboratory sterility testing and cell characterization. This clinical trial was designed to provide data for assessment of any impacts of the shipping and remote manufacturing.

## Materials and Methods

### Clinical study objectives

The primary objective of this study was to evaluate the safety of a single intra-articular dose of an investigational biologic product (IBP), PSC-01, an autologous, adipose-derived SVF for the treatment of KOA. The secondary objective was to obtain preliminary evidence of efficacy for PSC-01 in the target population. The data were submitted to the Food and Drug Administration (FDA) as part of the submission process to support potential regulatory approval.

### Study design

This study was designed as a single-arm, open-label, clinical safety study in subjects diagnosed with KOA. It was conducted at seven sites within the United States and with nine investigators experienced in orthopedics. The study was conducted in compliance with FDA regulations for phase 1/2A clinical trials and was approved by an Institutional Review Board.

### Eligibility

The target population included males and females, 18–80 years of age with Kellgren–Lawrence (K-L) grade 2, 3, or 4 in one knee, and at least weekly pain for a minimum duration of 3 months after failing conservative therapy. The subjects were included if they were otherwise healthy with no disease conditions that would impact safe participation in the study. Diagnosis was made with clinical and radiographic evaluation. Subjects were excluded if the contralateral knee had a K-L score greater than 2.

A total of 38 subjects were initially enrolled to achieve 29 subjects completing the study. Analgesics, NSAIDS, and supplements were allowed to be given during the study if the subject had been on that treatment for at least 30 days before enrollment and remained on that treatment at the same dose for the duration of the study. Steroids and injectable joint products were not allowed during the study or within 60 days before treatment.

### Adipose tissue harvest and SVF isolation

The IBP was an autologous, adipose-derived cell product extracted from adipose tissue by lipoaspiration (PSC-01, SVF). Subjects were screened, enrolled, and an adipose harvest conducted in the investigators clinic to acquire the tissue for extraction of the SVF cells. Following tumescent anesthesia with standard Klein's solution, a minimum of 100 mL lipoaspirate was targeted for collection by the investigator, decanted for 15 min, and then shipped to the central FDA-compliant, manufacturing cleanroom facility. The sample was shipped overnight on priority using Federal Express in a validated temperature-controlled shipping container to the Sponsor's facility. Before processing, a sample was taken for sterility assessment of the incoming sample to properly assess the source of any contamination. All lipoaspirate samples were processed within 24 h of collection.

In a cleanroom environment with biosafety cabinets, the lipoaspirate was enzymatically digested (collagenase, Nordmark Pharma GmbH), washed, centrifuged, and separated into a stromal vascular cell mixture and cryopreserved in one or more dose vials at the cGMP facility. Quality control (QC) samples were taken and stored in the same manner as the IBP doses for lot release assessment. All samples were stored in liquid nitrogen at <130°C, generally for 1–4 weeks before scheduled patient treatment. Storage stability testing has demonstrated stability for a minimum of 2 years. Our veterinary SVF research program has demonstrated frozen stability of SVF for >15 years.

### SVF assessment and lot release

After cryopreservation, the QC samples were removed from the storage freezers, thawed, and evaluated for cell count, viability, sterility, endotoxin, and by flow cytometry. In addition, the initial incoming sample was assessed for sterility to assess the collection quality and the shipping impacts.

The dose was determined by the total available SVF cells, with a minimum dose of 2 × 10^6^ nucleated cells and a maximum of 10 × 10^6^ nucleated cells. A cell counter (Nucleocounter by ChemoMetec, Denmark) using a validated propidium iodide cell counting method was used to measure the cell count and viability.

### SVF dose delivery

Once passing QC lot release criteria, the participant was scheduled for knee injection. The cells were shipped in a liquid nitrogen (<−130°C) dry shipper and stored on-site at the investigator's clinic until use with a safe storage period of 7 days after shipment. After thawing at room temperature, PSC-01 was injected under ultrasound guidance into the lateral suprapatellar recess of the selected joint with a 22 gauge or greater needle. A photograph of the procedure and ultrasound image were reviewed by the Sponsor medical director to confirm proper needle placement. Each participant received only a single dose of PSC-01 cells. No repeat dosing was allowed during the study. Subjects were permitted to bear weight as tolerated on the treated knee immediately following the procedure, and adjunct treatments were not used (e.g., physical therapy).

### Primary outcome safety

Collected safety assessment data included patient questionnaire, medical history, physical examination, vital signs, self-reported assessment, postlipoaspiration and postinjection observations, adverse events (AEs), and laboratory tests (complete blood count, blood chemistry panel, and urinalysis). An analysis of AEs and trends was conducted to determine the safety profile of the adipose harvest procedure and the IBP therapy. Particular attention was given to the days following adipose harvest and IBP injection for acute AEs. All treated subjects were followed poststudy for a 6-month and 12-month safety assessment. AEs were reported in terms of severity, resolution, and causality and were coded according to MedDRA (Medical Dictionary for Regulatory Activities) dictionary as to preferred term and SOC (MedDRA organ system classification).

### Efficacy evaluation

Treatment efficacy was measured with the Knee Injury and Osteoarthritis Outcome Score (KOOS). The KOOS is a knee-specific instrument, validated, clinically relevant, and reliable self-administered instrument that can be used for follow-up of several types of knee injury, including osteoarthritis [51]. The KOOS consists of 42 items in 5 separately scored subscales: Pain, Symptoms, Function in Daily Living (ADL), Function in Sport and Recreation (Sport), and knee-related Quality of Life (QOL). A Likert scale is used to answer 42 items with five possible options scored from 0 (No Problems) to 4 (Extreme Problems). Each subscale is calculated as the sum of the items. Scores are transformed to a 0–100 scale, with zero representing extreme knee problems and 100 representing no knee problems.

KOOS scores are often compared to a clinically relevant improvement. Roos and Lohmander [51] provided the logic for the use of this scoring paradigm to access changes following treatment over time in patients with KOA. These authors also recommended a minimal important clinical change (MIC) of 8–10 in the absence of a more refined MIC for a particular study or intervention. In our analysis, we used an MIC of 8. In another method to evaluate patients 2 years following TKA, Lyman proposed a minimal clinically important difference (MCID) for each of the KOOS subscales as 9, 8, 9, 8, and 6 for Pain, Symptoms, ADL, Sport, and QOL, respectively [52]. The KOOS was assessed at screening, day of treatment, interim follow-up visit, final visit (Day 84), and the 12-month time points.

To measure the subject's satisfaction at 12-month assessment, subjects were asked to respond to the question, “How satisfied are you with the results of the investigational stem cell treatment for your knee?” with a five-point Likert scale (very satisfied, satisfied, neutral, dissatisfied, or very dissatisfied).

## Results

### Demographics

A total of 37 subjects satisfied the screening criteria and underwent lipoaspiration. IBP release criteria were not met for eight subjects who were then withdrawn from the study. A total of 29 subjects received a single IBP injection with an average age of 65.6 years, average body mass index (BMI) of 27.5 kg/m^2^, and average K-L severity score of 2.9 with 21% grade 2, 69% grade 3, and 10% grade 4 (Table 1). Thirty-one percent (*N* = 9) of the subjects were male, 69% (*N* = 20) were female. All 29 participants injected with the IBP completed the study period with no withdrawals and completed all follow-up visits through 12 months.

**Table 1.** Baseline Data of Included Subjects

Average age (years)	Average BMI (kg/m^2^)	K-L severity score				Total participants treated
		Grade 2	Grade 3	Grade 4	Average grade	
65.6	27.5	21%	69%	10%	2.9	29

### SVF characterization

The average delivered dose in this study was 4.0 ± 1.8 × 10^6^ nucleated cells with an average viability post-thaw of 72.7% ± 7.1%. The average percentage regenerative cell composition (ASC and pericyte) was 24.3% of the total viable cells, and all delivered cells were no growth on sterility testing.

One patient sample had an incoming positive sterility result and that product was quarantined, and the patient removed from the study and not treated. This is a high value component of the central laboratory model when you know dose, sterility, and purity before patient treatment occurs.

### Safety outcomes

During the study period, no clinically significant changes in laboratory values, vital signs, BMI, or physical examination were identified following lipoaspiration or IBP injection.

Following lipoaspiration, AEs were assessed in all 37 subjects. A total of 13 grade 1 and 11 grade 2 AEs were reported. Of these, 17 were deemed related to the lipoaspiration procedure and included mild to moderate pain, bruising, subcutaneous hematoma, or numbness. All the reported AEs resolved before the end of the study without the need for ongoing treatment, and there were no serious AEs.

Following treatment with IBP injection, AEs were assessed in all 29 subjects. A total of 16 grade 1 and 15 grade 2 AEs were reported (Table 2). Of these, 6 were deemed related to the IBP injection and included mild to moderate pain or itching. All the reported AEs resolved before the end of the study without the need for ongoing treatment and there were no serious AEs.

**Table 2.** Adverse Events Reported During the Study by Body System Code (SOC) and Grade

Body system (SOC)	Grade 1		Grade 2		Grade 3		Grade 4		Total	
	R/RP	NR	R/RP	NR	R/RP	NR	R/RP	NR	R/RP	NR
Musculoskeletal and connective tissue disorders	1	5	1	9					2	14
General disorders and administration site conditions	3								3	0
Infections and infestations		1		1					0	2
Injury poisoning and procedural complications		1		1					0	2
Investigations		1		1					0	2
Nervous system disorders	1	1							1	1
Metabolism and nutrition				1					0	1
Endocrine disorders		1							0	1
Renal and urinary disorders		1							0	1
Surgical and medical procedures				1					0	1
Totals	5	11	1	14	0	0	0	0	6	25

R/RP, related/probably related; NR, not related.

### Poststudy safety outcomes

During the time period following the end of study (Day 84) to the 12-month follow-up, there were a total of nine grade 1 and eight grade 2 AEs, none of which was deemed related to the lipoaspiration or IBP treatment (Table 3). There were four grade 3 AEs reported, three of which were orthopedic issues not related to the treatment and one subject underwent a TKA due to continued osteoarthritis pain in the treated knee. All treatment-related AEs resolved before the 12-month follow-up, and there were no treatment-related serious AEs.

**Table 3.** Adverse Events Reported from Day 85 Through Month 12 by Body System Code (SOC) and Grade

Body system (SOC)	Grade 1	Grade 2	Grade 3	Grade 4	Total
Cardiac disorders		1			1
Gastrointestinal disorders		1			1
Musculoskeletal and connective tissue disorders	6	3	4		13
Neoplasms, benign, malignant, and unspecified		1			1
Nervous system disorders	2				2
Renal and urinary disorders		1			1
Respiratory, thoracic, and mediastinal disorders		1			1
Skin and subcutaneous tissue disorders	1				1
	9	8	4	0	21

### Efficacy outcomes

**Fig. 1.**
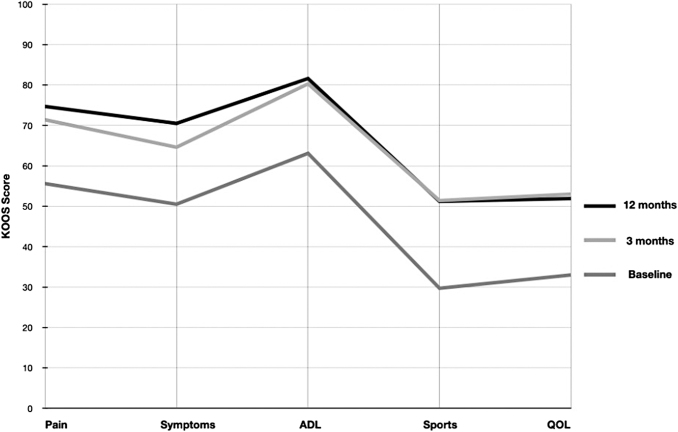
Improvement in Knee Injury and Osteoarthritis Outcome Score (KOOS) subscales (Pain, Symptoms, ADL, Sports and QOL) in response to treatment. Improvements were seen in all five KOOS subscales at 3 month followup (light gray) and maintained at 12 month followup (dark gray). Medium gray = baseline scores.

All 29 subjects completed the middle and end of study (Day 84) KOOS assessments. The baseline KOOS subscales were Pain (55.6), Symptoms (50.5), Daily Function (63.1), Sports (29.7), QOL (33.0), and average KOOS (52.5). All KOOS subscales improved at both the middle and end of study assessments with Pain (13.5 and 15.8), Symptoms (12.5 and 14.1), Daily Function (16.1 and 17.2), Sports (19.2 and 21.7), QOL (16.3 and 20), and average KOOS (15.4 and 17.8) noted in Table 4. At the end of the study, the mean of every KOOS subscale exceeded the recommended clinical MIC and MCID (Fig. 1).

**Table 4.** Changes in KOOS Subscale Scores

Category	*N*	Pain	Symptoms	Daily function	Sports	QOL	Average
Baseline	29	55.6	50.5	63.1	29.7	33.0	52.5
Scores at Day 84	29	71.4	64.6	80.3	51.4	53.0	69.8
Score changes from baseline to Day 84		15.8	14.1	17.2	21.7	20.0	17.3
% Improvement from baseline to Day 84		28.4%	28.0%	27.3%	73.3%	60.8%	32.9%
12-month follow-up	26	74.7	70.5	81.6	51.2	51.9	70.6
Score changes from baseline to 12 months		19.1	20.0	18.5	21.5	18.9	18.1
% Improvement from baseline to 12 months		34.4%	39.6%	29.3%	72.7%	57.5%	34.4%
% Improvement from day 84 to 12 months		4.7%	9.0%	1.5%	−0.3%	−2.1%	1.1%

QOL, quality of life.

### Poststudy efficacy outcomes

Three participants did not provide a KOOS evaluation as a result of having undergone knee joint replacement surgery, leaving a total of 26 participants for the 12-month poststudy efficacy analysis.

At 12 months, the mean of every KOOS subscale continued to exceed the suggested MIC. Compared to baseline, 79.3% of the subjects exceeded the average KOOS MIC. The proportion of subjects that exceeded the MIC for Pain, Symptoms, Daily Function, Sports, and QOL was 76%, 66%, 69%, 79%, and 76%, respectively.

At 12 months, the mean of every KOOS subscale continued to exceed the suggested MCID. Compared with the Day 84 assessment, the KOOS Pain, Symptoms, and ADL subscales all continued to improve at 12 months. The KOOS Sport and QOL subscales decreased slightly. The proportion of subjects that exceeded the MCID for Pain, Symptoms, Daily Function, Sports, and QOL was 69%, 66%, 69%, 79%, and 83%.

Furthermore, we evaluated the distribution of the KOOS outcomes by baseline radiographic severity (K-L grade) and baseline BMI. The proportion of subjects with an average KOOS exceeding the MIC for K-L grade 2, 3, and 4 was 83%, 85%, and 33%, respectively. The proportion of subjects with average KOOS exceeding the MIC for BMI rated as normal, overweight, and obese was 57%, 88%, and 83%, respectively.

There was no correlation between the proportion of subjects with an average KOOS exceeding the MIC and total nucleated cell count or with the percentage of adipose stem cells in the IBP.

At the 12-month mark, the majority of subjects (75.8%) were “very satisfied” or “satisfied” with their treatment. A total of 17.2% of participants were not satisfied with the treatment.

## Discussion

In this study, we explored the safety and efficacy of an autologous, adipose-derived SVF GMP-manufactured product in subjects with moderately severe KOA. Our findings are similar to other published clinical trials that reported on the safety of intra-articular SVF injection. Our subjects reported good procedure tolerability and only mild to moderate AEs. At 12-month follow-up, there were no serious treatment-related AEs, and most subjects were satisfied with their results. Functional and symptomatic improvements exceeded the suggested MCID at 3 months and persisted up to 12 months. Although our study did not include a control group, the magnitude of clinical improvement seen in this study is consistent with previously published clinical trials as shown in meta-analysis by Anil et al. [53].

To the best of our knowledge, this is the first US FDA-approved clinical trial of an autologous SVF product manufactured in an FDA-compliant, cGMP facility. The use of an autologous cell product enhances safety as there is no need to identify an HLA-matched donor and no risk of contamination from transmissible diseases or clonogenic tumor cells. Compliance with FDA cGMP guidelines assures the consistent production of an autologous cellular product with known identity, purity, and potency. Sterility testing and packaging further enhances product safety by minimizing the risk of propagation of pathogenic agents that might otherwise occur with point-of-care devices or other non-GMP manufacturing methods.

Furthermore, the question of degradation of viability of cells due to shipping was answered in that the viability of the central laboratory model SVF was equivalent to the literature reported viability for the same-day point-of-care methods while providing additional quality and safety testing [54,55].

Pain, stiffness, instability, and weakness are all seen in KOA patients because of degeneration and inflammation in both intra-articular and extra-articular joint tissues. Knee pain has been shown to correlate with the levels of inflammatory mediators such as IL-1β, IL-6, and TNF-α in the early stages of KOA [56]. Impaired regulation of angiogenesis in the synovium and osteochondral junction contributes to chronic inflammation, neo-innervation, and pain [57]. The involvement of multiple joint tissues complicates the treatment of this serious medical disease.

The infiltration of SVF into a pro-inflammatory environment can activate ASCs to modulate immune cells mainly through the production of IL-1Ra, IDO, IL-4, IL-10, prostaglandin 2, and TGF-β [45]. Polarization of macrophages to anti-inflammatory type M2 would express IL-4, IL-10, and IGF-1 and inhibit production of TNF-α. This has the effect of reducing metalloproteinase levels stopping the pro-degenerative effects and restoring tissue homeostasis.

The present study has some limitations. First, we did not include a placebo group as our intention was to measure safety. Furthermore, we included a small number of subjects. Future investigations on SVF efficacy will require larger samples to measure effect size. Long-term follow-up would be warranted to fully understand the durability of this treatment.

## Conclusions

KOA is a serious medical condition for which there are no treatments offering long-term symptomatic relief. We were able to demonstrate good tolerability, safety profile, and preliminary efficacy at 12 months with a single injection of autologous, adipose derived, cGMP manufactured SVF in subjects with moderately severe KOA. The results of this study are consistent with previously reported safety and efficacy in a number of published clinical trials. These authors believe that the unique approach of providing a central cGMP laboratory derived autologous cell therapy, with cells stored for future possible treatments, is worthy of further development. This scientific evidence provides strong support for the development of a placebo-controlled, randomized clinical trial with long-term follow-up.
